# Characterisation of preproendothelin-1 derived peptides identifies Endothelin-Like Domain Peptide as a modulator of Endothelin-1

**DOI:** 10.1038/s41598-017-05365-2

**Published:** 2017-07-10

**Authors:** Jale Yuzugulen, Julie A. Douthwaite, Elizabeth G. Wood, Inmaculada C. Villar, Nimesh S. A. Patel, James Jegard, Hubert Gaertner, Irène Rossitto-Borlat, Keith Rose, Oliver Hartley, Pedro R. Cutillas, Amrita Ahluwalia, Roger Corder

**Affiliations:** 1 William Harvey Research Institute, Barts and the London School of Medicine and Dentistry, Queen Mary University of London, London, EC1M 6BQ UK; 2 Barts Cancer Institute, Barts and the London School of Medicine and Dentistry, Queen Mary University of London, London, EC1M 6BQ UK; 3 Faculty of Medicine, University of Geneva, 1211 Geneva 4, Switzerland

## Abstract

Endothelin-1 (ET-1) is involved in the pathogenesis of cardiac and renal diseases, and in the progression of tumour growth in cancer, but current diagnosis and treatment remain inadequate. Peptides derived from the 212 amino acid precursor preproendothelin-1 (ppET-1) may have utility as biomarkers, or cause biological effects that are unaffected by endothelin receptor antagonists. Here, we used specific immunoassays and LC-MS/MS to identify NT-proET-1 (ppET-1_[18–50]_), Endothelin-Like Domain Peptide (ELDP, ppET-1_[93–166]_) and CT-proET-1 (ppET-1_[169–212]_) in conditioned media from cultured endothelial cells. Synthesis of these peptides correlated with ET-1, and plasma ELDP and CT-proET-1 were elevated in patients with chronic heart failure. Clearance rates of NT-proET-1, ELDP and CT-proET-1 were determined after *i.v*. injection in anaesthetised rats. CT-proET-1 had the slowest systemic clearance, hence providing a biological basis for it being a better biomarker of ET-1 synthesis. ELDP contains the evolutionary conserved endothelin-like domain sequence, which potentially confers biological activity. On isolated arteries ELDP lacked direct vasoconstrictor effects. However, it enhanced ET-1 vasoconstriction and prolonged the increase in blood pressure in anaesthetised rats. ELDP may therefore contribute to disease pathogenesis by augmenting ET-1 responses.

## Introduction

The 21-amino acid vasoconstrictor peptide endothelin-1 (ET-1) was first described almost 30 years ago^1^. Subsequent research has strongly linked increased expression of ET-1 to pathological processes underlying cardiovascular, renal and lung diseases^2–6^, where it not only acts as a vasoconstrictor but also causes inflammation, vascular remodelling and fibrosis^7–13^. ET-1 peptides also play a role in cancer cell proliferation and tumour growth^14^. Current use of endothelin receptor antagonists (ERA) is mainly restricted to treatment of pulmonary hypertension^5, 6^. Other disease areas, particularly those involving fibrosis or tumour growth, are largely resistant to ERA treatment^14^. However, observations that over expression of the *EDN1* gene leads to a lethal heart failure that is not prevented by ERA indicate that there are undiscovered aspects of endothelin biology that require further investigation^7^.

There are three endothelin isoforms – ET-1, ET-2 and ET-3 – generated from three separate genes^15^. ET-1 is the main isoform expressed in the cardiovascular system. Human ET-1 is derived from a 212 amino acid precursor – preproET-1 (ppET-1)^16, 17^. All three isoforms are 21 amino acids long and contain four Cys residues that form two intra-chain disulphide bridges at Cys^1^–Cys^15^ and Cys^3^–Cys^11^ ^1, 15^. This unusual structure is an evolutionary conserved characteristic of this family of peptides^18, 19^.

Processing of ppET-1 occurs intracellularly^20^. This involves removal of the N-terminal signal sequence, predicted to be preproET-1_[1–17]_^16, 17^, by a signal peptidase in the endoplasmic reticulum as the nascent ET-1 precursor enters the secretory pathway^21^. This generates proET-1 (ppET-1_[18–212]_), which enters the constitutive secretory pathway via the *trans* Golgi network and is then transported to the cell surface in constitutive secretory vesicles^20^. Processing of proET-1 to ET-1 occurs in transit to the cell surface, following a pattern consistent with other biologically active peptide prohormones. This involves specific proteolytic cleavage on the C-terminal side of double basic amino acid residues (most commonly at Lys-Arg and Arg-Arg) by prohormone/proprotein convertases, and is followed by removal of C-terminal basic residues by carboxypeptidase H^22^. Cleavage at double basics residues generates the inactive intermediate bigET-1, which is converted intracellularly by endothelin-converting enzyme to ET-1^20^. Processing of proET-1 at double basic residues yields additional peptide fragments that are co-secreted with the biologically active ET-1 from endothelial cells and other cells synthesising ET-1^23^. These peptide fragments are generally considered to be biologically inert, but can act as biomarkers of ET-1 release^24, 25^.

**Figure 1:**
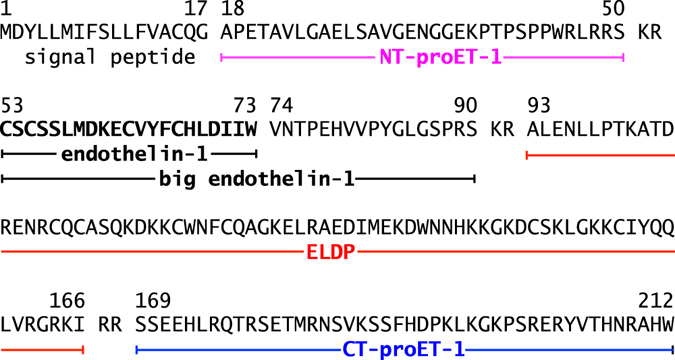
Amino acid sequence of preproendothelin-1 indicating the peptide products generated by processing at double basic residues.

Here we describe the detailed characterisation of the peptide products of intracellular processing of proET-1 – namely, NT-proET-1 (ppET-1_[18–50]_), Endothelin-Like Domain Peptide (ELDP, ppET-1_[93–166]_) and CT-proET-1 (ppET-1_[169–212]_) (Fig. 1). To assess their relative utility as biomarkers of disease, plasma concentrations in patients with chronic heart failure were compared to subjects free of symptoms of heart disease. To determine whether metabolism in the circulation influenced plasma levels of these peptide fragments relative clearance rates were measured in anaesthetised rats after *i.v*. injection. Finally, given the structural homology to ET-1, we examined whether ELDP displayed vasoconstrictor activity or modified the effects of ET-1.

## Results

### Initial characterisation of secreted proET-1 derived peptides

**Figure 2:**
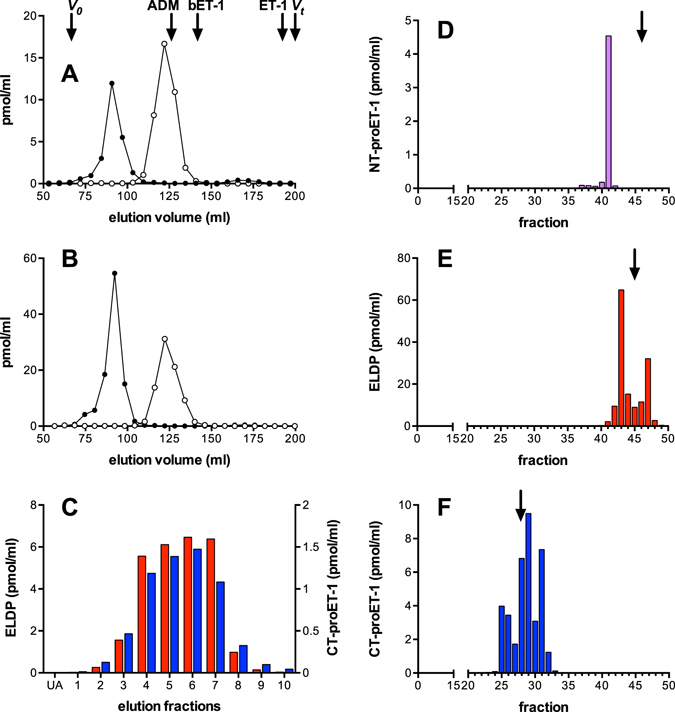
Size exclusion chromatography of peptide precipitates from (**A**) EA.hy 926 and (**B**) A549 cell lines, (**C**) cation exchange chromatography of conditioned media from EA.hy 926, and HPLC characterisation of pro-ET-1 peptides from EA.hy 926 cells – (**D**) NT-proET-1, (**E**) ELDP and (**F**) CT-proET-1. (**A**) and (**B**) proendothelin-1 peptides from EA.hy 926 and A549 cell lines were subjected to size exclusion chromatography (column 1.6 × 100 cm, Sephadex G50 superfine) equilibrated at 4 °C with 0.1 M NaCl containing 0.01 M HCl. Fractions were subjected to immunoassay for ppET-1_[42–50]_ (open circles) and ppET-1_[93–102]_ (closed circles). Elution positions of adrenomedullin (ADM, *M*_*r*_ 6029), big ET-1 (bET-1, *M*_*r*_ 4283) and ET-1 (*M*_*r*_ 2492) are indicated. (**C**) Cation-exchange chromatography of 300 ml of acidified (1.25% acetic acid) conditioned medium from EA.hy 926 cells loaded onto ten Fractogel CEx columns (bed volume 2.8 ml each) after Q-Sepharose FF pre-treatment. After rinsing with 10 mM acetic acid (10 ml/column) to remove unadsorbed material (UA), columns were eluted sequentially (3 ml each) with: (1) 0.125 M NaCl, (2) 0.25 M NaCl, (3) 0.25 M NaCl + 0.1 M G-HCl, (4) 0.5 M NaCl + 0.1 M G-HCl, (5) 1 M NaCl + 0.1 M G-HCl, (6–10) 1 M NaCl + 0.5 M G-HCl. ELDP (red columns) and CT-proET-1 (blue columns) were measured using immunoassays. (**D**), (**E**) and (**F**) peak fractions of NT-proET-1, ELDP and CT-proET-1 from semi-preparative RP-HPLC were pooled and subjected to separate RP-HPLC purification (ACE-5 C4 300 Å, 5 µm, 4.6 × 250 mm) with gradient elution (1 ml/min) 0.1% TFA – 10% solvent B (80% CH_3_CN with 0.1% TFA) over 2 min, followed by 10–30% B over 50 min. Fractions (1 min) were subjected to specific sandwich immunoassay to identify peak fractions for LC-MS/MS characterisation. Arrows mark the elution positions of synthetic NT-proET-1, ELDP and CT-proET-1; which eluted in fractions 46, 45 and 28, respectively.

To identify the peptide products generated by intracellular processing of proET-1, two ET-1 producing cell lines were used: EA.hy 926 and A549^26–28^. EA.hy 926 is a human hybridoma cell line with characteristics of endothelial cells^26^, which was created by fusing human umbilical vein endothelial cells with A549 cells (a human lung carcinoma cell line with epithelial cell characteristics^27^). Initial characterisation focused on identifying the peptides produced on cleavage of the double basic residues flanking big ET-1. To achieve this ammonium sulphate peptide precipitates were prepared from conditioned media collected after 48 h incubation with EA.hy 926 and A549 cell lines, and subjected to size exclusion chromatography on Sephadex G50 (Fig. 2). Immunoassay of fractions for ppET-1_[42–50]_ identified a single peptide peak from both cell lines. The estimated molecular weight was ≈6 kD based on elution of reference peptides (Fig. 2A and B), compared to its theoretical mass of 3430. This anomaly may be due to NT-proET-1 having a larger hydrodynamic radius than the reference peptides, which all contained disulphide bonds and likely have more compact structures. Nevertheless, this indicated that NT-proET-1 is synthesised as the full-length peptide resulting from removal of the signal peptide and processing at Lys^51^-Arg^52^ (Fig. 1).

Immunoassay for ppET-1_[93–102]_ also identified a signal peak (Fig. 2A and B) from both cell types. This corresponded to a molecular weight of ≈8–9 kD, representing a long internal sequence of proET-1. Based on the size of the peptide it was hypothesised that processing at double basic residues occurred at Arg^164^-Lys^165^ or Arg^167^-Arg^168^ (Fig. 1). If processing occurred at the earlier of these paired residues the resulting C-terminal Gly^163^ would likely be converted to Arg^162^-amide by peptidylglycine alpha-amidating monooxygenase^29^. Therefore, to identify the C-terminal of this peptide, antisera were raised against ppET-1_[155–162-amide]_ and ppET-1_[155–166]_. Native peptide in conditioned media samples was only recognised by ppET-1_[155–166]_ antiserum, which indicated that this large peptide from the mid-portion of proET-1 was ppET-1_[93–166]_ (theoretical mass 8637), which we called Endothelin-Like Domain Peptide (ELDP) because of the evolutionary conserved endothelin-like domain^18, 19^.

Based on this initial characterisation of proET-1 derived peptides in these conditioned media samples we hypothesised that the following peptides were generated by proET-1 processing – NT-proET-1 (ppET-1_[18–50]_), ELDP (ppET-1_[93–166]_) and CT-proET-1 (ppET-1_[169–212]_) (Fig. 1). To confirm this we developed double-recognition site sandwich immunoassays using peptide antigens that represented the N- and C-terminal sequences for each of these peptides (Table 1).

**Table 1:** Antibodies used for proendothelin-1 sandwich immunoassays.

ProET-1 peptide sequences	Capture Ab/Protein Conjugate	Species	Detection Ab/Protein Conjugate	Species
**NT-proET-1** (ppET-1_[18–50]_): **APETAVLGAELSAV**GENGGEKPTP**SPPWRLRRS**	ppET-1_[18–30]_/Maleimide	Rabbit	ppET-1_[42–50]_/Glutaraldehyde	Rabbit
**ELDP** (ppET-1_[93–166]_): **ALENLLPTKATDRENRC**QCASQKDKKCWNFCQAGKELRAEDIMEKDWNNHKKGKDCSKLGKK**CIYQQLVRGRKI**	ppET-1_[93–109]_/Maleimide	Sheep	ppET-1_[155–166]_/Maleimide	Sheep
**CT-proET-1** (ppET-1_[169–212]_): **SSEEHLRQTRSETMRNSV**KSSFHDPKLKGKPSRER**YVTHNRAHW**	ppET-1_[169–186]_/Maleimide	Sheep	ppET-1_[204–212]_/Glutaraldehyde	Rabbit

Amino acid numbering is based on the 212 amino acid sequence for preproendothelin-1 (ppET-1). Underlined sequences in bold type indicate peptides used as antigens to raise specific antisera for the development of capture and detection antibodies.

### Purification and LC-MS/MS characterisation of proET-1 peptides

ELDP and CT-proET-1 were isolated from conditioned media collected from EA.hy 926 cells by cation-exchange (Fractogel CEx) chromatography after passing the acidified media through a strong anion exchange gel (Q-Sepharose FF). The elution characteristics of these peptides were typical of their high isoelectric points (ELDP pI 9.4; CT-proET-1 pI 10.5) (Fig. 2C). NT-proET-1 (pI 6.3) immunoreactivity was mainly present in the unadsorbed pooled sample that had passed through both Q-Sepharose FF and Fractogel CEx. Subsequent isolation of NT-proET-1 immunoreactivity, initially by extraction from this unadsorbed material on C2-silica cartridges, resulted in a single peak on reverse-phase HPLC, which eluted several fractions earlier than synthetic standard (Fig. 2D). Trp oxidation or deamidation of Asn during the isolation process likely accounts for the reduced hydrophobicity of native NT-proET-1. Based on antibody crossreactivity, and characteristics on ion-exchange chromatography and RP-HPLC, it seems likely that purified NT-proET-1 corresponded to the predicted amino acid sequence ppET-1_[18–50]_^16, 17^. However, there was insufficient purified peptide for confirmation of this by UPLC–MS/MS, despite pre-optimization of the elution conditions using synthetic NT-proET-1.

Purification of ELDP and CT-proET-1 by HPLC revealed elution characteristics of the purified native peptides very similar to synthetic peptide standards (Fig. 2E and F). The slight heterogeneity of both purified peptides, based on elution profile, was likely due to oxidation (His, Met, or Trp), and side chain hydrolysis of Asn and Gln during incubation in conditioning media prior to collection or as a consequence of the purification conditions (these modifications were observed on LC-MS/MS, Supplementary Tables S1 and S2). To enable detection of peptides with intact disulphide bridges trypsin digestion was performed without reduction or alkylation. Partial amino acid sequence was obtained for these purified peptides by LC-MS/MS. For ELDP, the peptides identified covered the ppET-1_[128–144]_ and ppET-1_[155–162]_ sequences (Supplementary Table S1, and Supplementary Figure S1), but no peptides containing disulphide bridges were detected. Analysis of synthetic ELDP following the same protocol also failed to identify the disulphide bridge structures, indicating the technical limitations of this approach. The endothelin-like domain sequence is rich in Lys, with four potential cleavage sites. Manual inspection of MS/MS data for all potential products of trypsin hydrolysis failed to identify any relevant peptides, suggesting that these relatively hydrophilic peptides were lost during sample preparation.

For purified CT-proET-1, amino acid sequences were identified that covered the majority of the proposed sequence (ppET-1_[169–212]_). Importantly, this included identification of the N-terminal sequence of CT-proET-1, ppET-1_[169–183]_ SSEEHLRQTRSETMR (Supplementary Table S2, and Supplementary Figure S2). When considered in conjunction with antibody recognition of ppET-1_[155–166]_, and LC-MS/MS identification of CIYQQLVR (ppET-1_[155–162]_) from the C-terminal sequence of ELDP, it can be concluded that ELDP is not processed to amidated ppET-1_[93–162-amide]_ (Supplementary Table S1, and Supplementary Figure S1). Hence confirming that Arg^167^-Arg^168^ is the ppET-1 processing site (_155_-CIYQQLVRGRKI**RR**SSEEHLRQTRSETMR-_183_). Antibody recognition by sandwich ELISA and elution characteristics of synthetic and purified native peptides, also strongly support the identities of ELDP and CT-proET-1 as ppET-1_[93–166]_ and ppET-1_[169–212]_.

### Evaluation of proET-1 peptides as biomarkers of ET-1 synthesis

**Figure 3:**
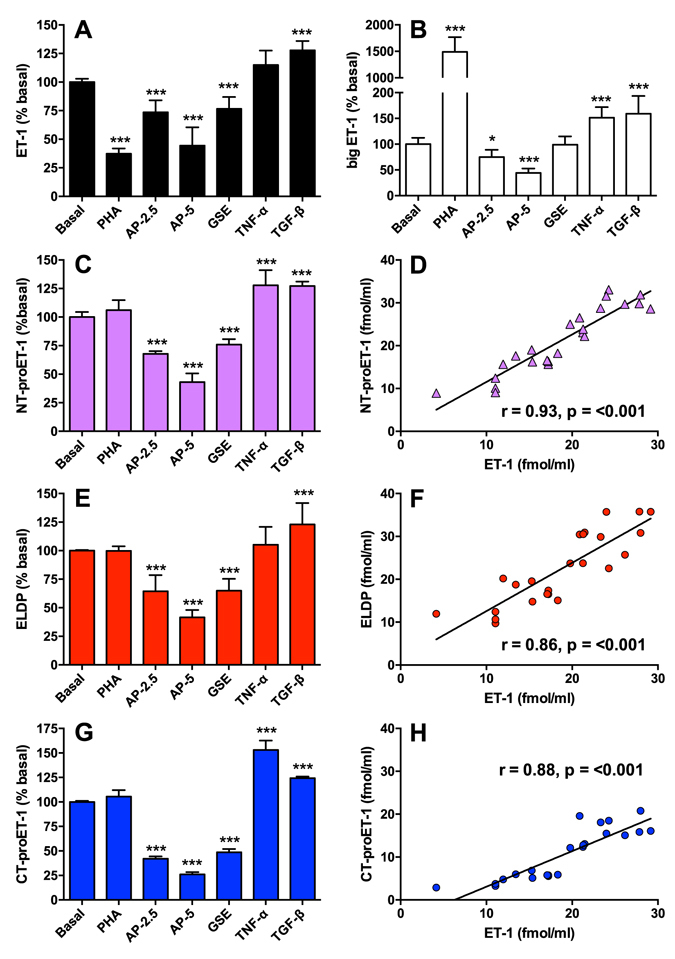
Correlation between secretion of ET-1 and proET-1 peptides from human aortic endothelial cells. Confluent cultures were incubated for 6 h in basal medium alone, or with 1 mM phosphoramidon (PHA), 2.5 or 5 µg/ml apple procyanidin pentamer (AP-2.5, AP-5), 5 µg/ml grape seed extract (GSE), 10 ng/ml TNFα or 1 ng/ml TGFß. All peptides were measured using specific sandwich ELISAs, and converted to % basal release (**A**,**B**,**C**,**E** and **G**). Results are mean ± SD from 4 experiments. Significant differences were determined by ANOVA with Bonferroni correction for multiple comparisons (*P = < 0.05; ***P = < 0.001). (**D**,**F**) and (**H**) show correlations between secretion of ET-1 and the respective proET-1 peptides under the different incubation conditions (excluding data obtained with phosporamidon).

To confirm that proET-1 peptides are a reproducible index of ET-1 synthesis we compared the level of peptide production in cultured human aortic endothelial cells under conditions known to increase or decrease ET-1 expression. These experiments also confirmed that early passage human endothelial cells from primary cultures produce the same peptides with the stoichiometric relationship expected for proET-1 processing. Treatment with phosphoramidon (PHA, 1 mM), an endothelin-converting enzyme inhibitor which blocks processing of big ET-1 to ET-1 without changing the overall level of proET-1 synthesis^30^, suppressed ET-1 synthesis with a consequent 14.9 ± 2.8 fold increase in big ET-1 compared to basal (Fig. 3). PHA had no effect on proET-1 peptide production showing that inhibition of endothelin-converting enzyme does not affect processing at double basic residues.

Apple procyanidin pentamer (AP 2.5 or 5 μg/ml) and a procyanidin-rich grape seed extract, which cause concentration dependent suppression of *EDN1* gene expression^31, 32^, reduced the synthesis of all peptides. Stimulation with TNFα (10 ng/ml) increases *EDN1* mRNA levels^33^. Consistent with this, levels of NT-proET-1 and CT-proET-1 in conditioned media samples significantly increased. ET-1 and ELDP did not change, which may indicate increased clearance of the secreted peptides due to TNF-induced peptidase activity or receptor-mediated clearance. This may be further compounded by reduced processing efficiency of proET-1, as big ET-1 concentrations increased with TNFα, so that increased mRNA levels were not reflected by increased secretion of ET-1 and ELDP. In comparison, TGFß significantly increased secretion of ET-1, big ET-1 and all the proET-1 peptides (Fig. 3). After excluding measurements made in the presence of PHA, ET-1 output was closely correlated with synthesis of all proET-1 peptides (Fig. 3), indicating their potential utility as circulating biomarkers that reflect ET-1 synthesis.

**Figure 4:**
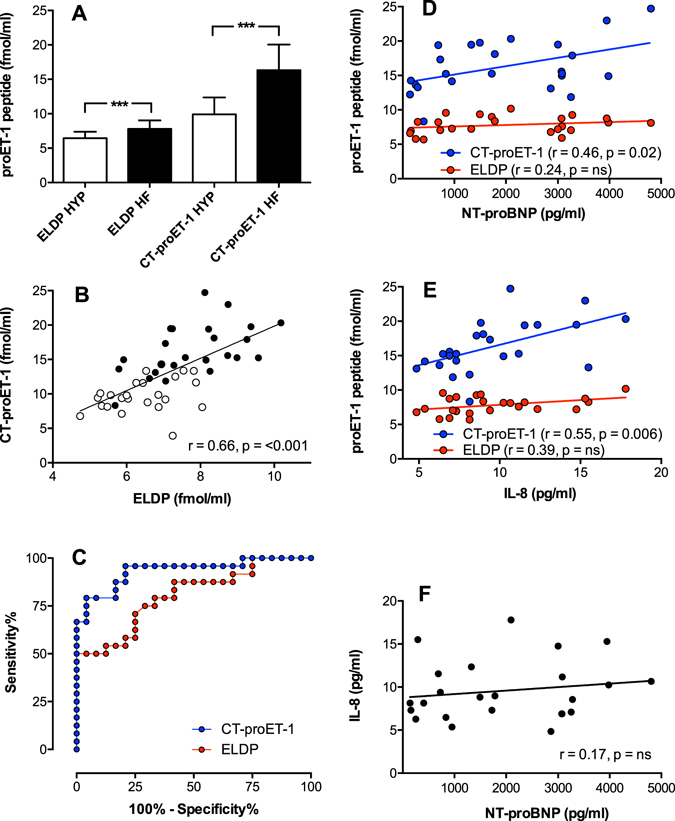
Comparison of plasma levels of ELDP and CT-proET-1 in hypertension and chronic heart failure. (**A**) Concentrations of ELDP and CT-proET-1 (mean ± SD) in each group (n = 24 per group) were compared by Student’s t-test, ***p < 0.001. (**B**) Correlation between individual plasma levels of ELDP and CT-proET-1 (open circles patients with hypertension, closed circles patients with chronic heart failure). (**C**) Comparison of ROC analyses for ELDP and CT-proET-1 detection of chronic heart failure. (**D**) and (**E**) Correlation between individual plasma levels of ELDP (red circles) and CT-proET-1 (blue circles) with NT-proBNP and IL-8 in patients with chronic heart failure. (**F**) Comparison of plasma levels of NT-proBNP and IL-8 in chronic heart failure.

To compare the relative potential of the three proET-1 peptides as biomarkers of cardiovascular disease, plasma levels were measured in patients with chronic heart failure due to ischemic heart disease and compared to control subjects with pre-hypertension/mild hypertension who were free of symptoms of heart disease (Table 2). Assay of NT-proET-1 failed to detect this peptide in plasma samples. Both ELDP and CT-proET-1 were significantly higher in samples from patients with chronic heart failure versus those with pre-hypertension/mild hypertension (p < 0.001) (Fig. 4A). Consistent with the stoichiometric co-synthesis of these peptides with ET-1, concentrations of ELDP correlated with CT-proET-1 (p < 0.001, r = 0.66) (Fig. 4B), which adds further support for these peptides being an index of ET-1 expression. CT-proET-1 concentrations were ≈80% greater than the corresponding ELDP values (p < 0.001; mean ± SD: ELDP 7.1 ± 1.3, CT-proET-1 13.1 ± 4.5 fmol/ml), indicating that clearance or metabolism of these peptides may differ.

**Table 2:** Patient information.

	Chronic Heart Failure	Hypertension
n	24	24
Age (years)	70 ± 2.0	55.4 ± 1.5
Male gender	20 (80%)	24 (100%)
Weight (kg)	85.7 ± 4.3	80.4 ± 3.0
Body Mass Index (kg/m^2^)	29.6 ± 1.2	26.7 ± 0.5
Systolic Blood Pressure (mmHg)	126.3 ± 3.6	144.8 ± 2.1
Diastolic Blood Pressure (mmHg)	71.0 ± 2.0	87.1 ± 1.3
Heart rate (bpm)	64.0 ± 1.4	69.3 ± 2.0
NYHA chronic HF Class II/III	20 (80%)/4 (20%)	—
LVEF (%)	31.2 ± 2.0	—
NT-proBNP (pg/ml)	1970 ± 288	—
CRP (mg/L)	4.05 ± 3.60	—
IL-6 (pg/ml)	2.90 ± 1.05	—
IL-8 (pg/ml)	9.58 ± 3.46	—
MCP-1 (pg/ml)	56.0 ± 22.7	—
TNFα (pg/ml)	3.12 ± 1.31	—
Total cholesterol (mmol/L)	4.25 ± 0.24	5.73 ± 0.20
LDL-cholesterol (mmol/L)	2.14 ± 0.25	3.53 ± 0.15
HDL-cholesterol (mmol/L)	1.29 ± 0.07	1.58 ± 0.06
Triglycerides (mmol/L)	1.75 ± 0.18	1.39 ± 0.11

It is already recognised that measurements of CT-proET-1 predict outcome in patients with chronic heart failure^34, 35^. To confirm that CT-proET-1 was a better a biomarker than ELDP for evaluating the severity of chronic heart failure, values for the two assays were compared using ROC analyses (Fig. 4C). Table 3 shows the area under the curves (AUC) for the two assays, and the sensitivity and specificity at various cutoffs – where sensitivity is the proportion of patients with chronic heart failure with a value greater than the cut-off, and specificity is the proportion of controls below this level. Both ELDP and CT-proET-1 showed good discrimination for patients with heart failure from individuals without heart disease (Table 3). The AUC for CT-proET-1 was significantly greater than ELPD (0.934 compared to 0.805, P = 0.019), indicating that it was a more sensitive biomarker and most likely to have diagnostic utility. For CT-proET-1 75.0% of patients with heart failure had values >13.89 fmol/ml and 95.8% of control subjects were below this level.

**Table 3:** ROC analyses for sensitivity and specificity of plasma assays of ELDP and CT-proET-1 for detection of chronic heart failure.

Concentration (fmol/ml)	Sensitivity (%)	Specificity (%)	AUC (95% CI)
ELDP			0.805 (0.682 to 0.927)
			P = 0.0003
>4.98	100.0	4.2	
>5.63	100.0	25.0	
>5.74	95.8	25.0	
>5.90	91.7	33.3	
>6.70	83.3	58.3	
>7.00	75.0	70.8	
CT-proET-1			0.934 (0.863 to 1.005)
			P = <0.0001
>5.355	100.0	4.2	
>8.300	100.0	29.2	
>11.77	95.8	79.2	
>12.05	91.6	79.2	
>13.20	83.3	83.3	
>13.89	75.0	95.8	

Consistent with previous biomarker studies in chronic heart failure where CT-proET-1 correlated with B-type natriuretic peptide (BNP)^34^, and log CT-proET-1 correlated with log N-terminal-proBNP (NT-proBNP)^35^, here CT-proET-1 correlated with NT-proBNP (Fig. 4D). A number of biomarkers of inflammation are elevated in chronic heart failure including IL-8, which is an independent predictor of outcome^36^. In this group of patients with stable chronic heart failure IL-8 correlated with CT-proET-1 (Fig. 4E) even though IL-8 did not correlate with NT-proBNP (Fig. 4F). This suggests that CT-proET-1 reflects both the level of inflammation and the degree of myocardial dysfunction in chronic heart failure. In agreement with ELDP being a less sensitive biomarker than CT-proET-1, its correlation with NT-proBNP or IL-8 did not reach statistical significance. In comparisons with other inflammation biomarkers, neither CT-proET-1 nor ELDP correlated with CRP, IL-6, MCP-1 or TNF (for all r ≤ 0.19 and p ≥ 0.4).

### Comparison of proET-1 peptide clearance rates

**Figure 5:**
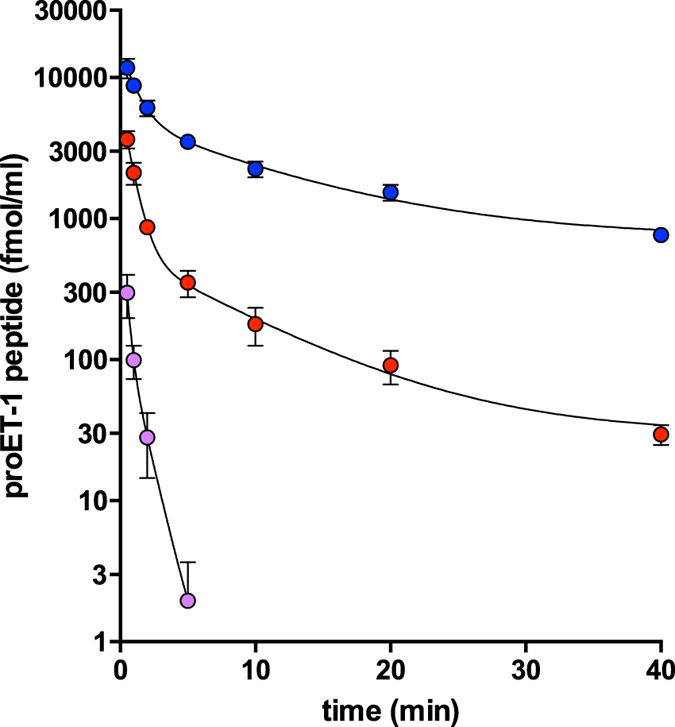
Comparison of clearance rates of NT-proET-1, ELDP and CT-proET-1 in anaesthetised rats. A bolus dose containing 1 nmol/kg of each proET-1 peptide as a combined solution prepared in 0.9% saline/0.1% BSA was administered *i.v*. and heparinised arterial blood samples (0.5 ml) were collected at 0.5, 1, 2, 5, 10, 20 and 40 min after administration of proET-1 peptides. NT-proET-1 (mauve circles), ELDP (red circles) and CT-proET-1 (blue circles) were measured by magnetic bead-based multiplex assays using Luminex methodology (values are mean ± SD, except where SD is less than the symbol size). Clearance rates for the three peptides were significantly different from each other (2-way ANOVA, P = < 0.001).

To examine whether circulating half-lives influenced plasma levels of proET-1 peptides clearance rates were investigated in anaesthetised rats. Bolus administration (1 nmol/kg *i.v*. of each peptide as a combined solution) showed markedly different elimination rates for NT-proET-1, ELDP and CT-proET-1 (p < 0.001; Fig. 5). Based on a circulating blood volume of 7.2 ± 0.3 ml per 100 g body weight^37^, a mean weight of 345 ± 6 g and hematocrit of 40–60%, the hypothetical maximum plasma concentrations were 23–35 pmol/ml. However, the levels of NT-proET-1, ELDP and CT-proET-1 30 s after injection were 0.29 ± 0.02, 3.65 ± 0.23, and 11.74 ± 0.64 pmol/ml, respectively. Relative to estimates for dilution in circulating blood, these values represent ≈1%, 10–16%, and 34–51% of administered peptide for NT-proET-1, ELDP and CT-proET-1. Moreover, NT-proET-1 was almost completely cleared from the circulation in <5 min (estimated initial t_½_ < 20 s). Clearance rates for ELDP and CT-proET-1 showed two phases with estimated t_½_ values of 0.5 and 0.7 min for phase 1, and 5.7 and 7.3 min for phase 2, with CT-proET-1 having significantly slower clearance than ELDP (P < 0.001) (Fig. 5).

### Cardiovascular effects of ELDP

**Figure 6:**
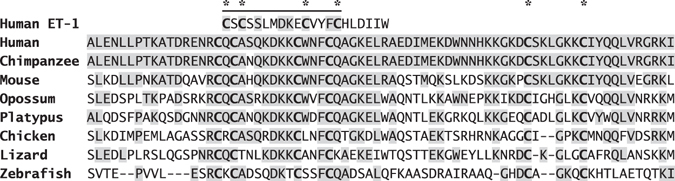
Inter species comparison of the conserved *EDN1* gene derived endothelin-like domain peptide (ELDP) with ET-1. The single line indicates the endothelin-like domain, with alignment of human ELDP and ET-1. Shaded residues indicate homology with human ELDP, and asterisks indicate conserved Cys residues. Peptide sequences are from the UniProt KnowledgeBase: human (*Homo sapiens*, P05305), chimpanzee (*Pan troglodytes*, H2QSB1), mouse (*Mus musculus*, P22387), opossum (*Monodelphis domestica*, F6YR31), duckbill platypus (*Ornithorhynchus anatinus*, F6WZM8), chicken (*Gallus gallus*, F1NWA9), green anole lizard (*Anolis carolinensis*, H9GDX1), and zebrafish (*Danio rerio*, A5WW41).

The endothelin-like domain sequence is highly conserved with the first four Cys residues having identical spacing to ET-1 (Fig. 6)^18, 19^. There is also considerable sequence homology adjacent to the endothelin-like domain, which has been retained throughout mammalian evolution, such that human ppET-1_[107–131]_ has 92% sequence identity with mouse ELDP and 88% with platypus over this sequence. Although this is suggestive of biological activity, previous attempts to demonstrate this for ppET-1_[110–130]_ failed to show any cardiovascular effects^38^. However, choice of test conditions and peptide structure may be critical for revealing the actions of ELDP. The C-terminal sequence of ELDP may be required for receptor binding, and hence for biological activity, as there are two additional Cys residues (ppET-1 Cys^148^ and Cys^155^) (Fig. 6), which have retained a consistent spacing from platypus to human, with several adjacent residues in the C-terminal sequence of ELDP (ppET-1_[143–166]_) being fully conserved (Lys^143^, Lys^144^, Gly^152^, Lys^154^, Gln^159^, Leu^160^, Val^161^ and Lys^165^) and others being mainly conservative substitutions.

**Figure 7:**
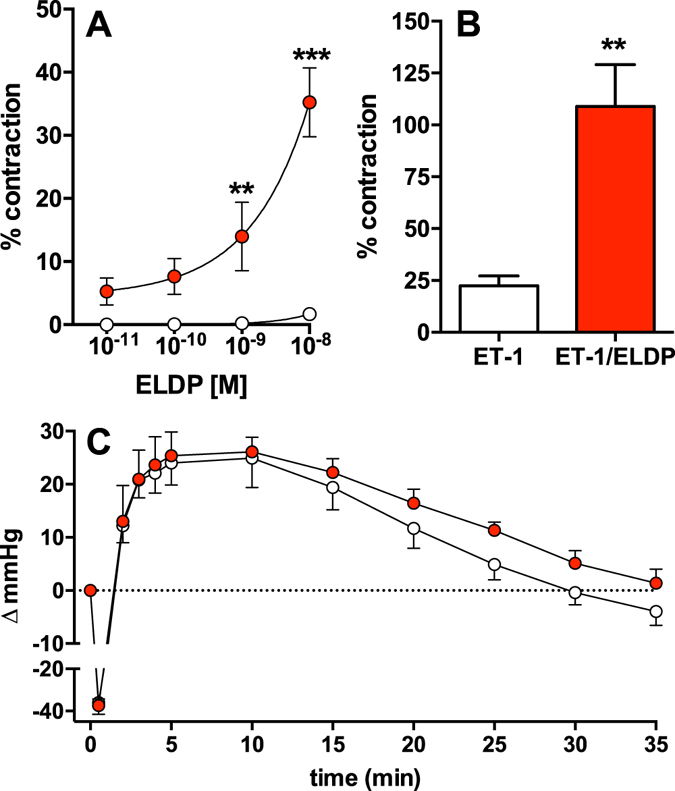
Cardiovascular effects of ELDP. (**A**) and (**B**) isolated rat mesenteric resistance arteries; (**A**) effect of ELDP_[162-amide]_ alone (white circles, n = 12) or after pre-treatment with ET-1 (1–3 × 10^−9^ M) to induce ≈10% vasoconstriction relative to the maximal response to phenylephrine (10 µM) (red circles, n = 7) (**P < 0.01, ***P < 0.001, compared to the same concentration without ET-1; 1 × 10^−8^ M ELDP_[162-amide]_ with ET-1 caused a significantly greater vasoconstriction than all other concentrations of ELDP_[162-amide]_ with ET-1, P < 0.001; 2-way ANOVA with Bonferroni correction). (**B**) vasoconstrictor response to ET-1 (1 × 10^−9^ M) alone (n = 8) or after pre-incubation with ELDP_[162-amide]_ (10 × 10^−9^ M, n = 10); P < 0.002; Student’s t-test. (**C**) Change in mean arterial blood pressure in anaesthetised rats following ET-1 (0.3 nmol/kg, *i.v*.) alone (white circles, n = 6) or 15 min after ELDP (3 nmol/kg *i.v*.) (red circles, n = 6). Following ELDP the response to ET-1 was significantly greater; P < 0.02, by 2-way ANOVA. Results are mean ± s.e.m.

Here, ELDP_[162-amide]_ alone (up to 10 nM) had no significant vasoconstrictor effect on rat mesenteric resistance arteries. However, after pre-treatment with ET-1 (1–3 nM) to induce a degree of vascular tone (≈10% vasoconstriction), ELDP_[162-amide]_ produced a concentration-dependent vasoconstriction (Fig. 7A). In addition, pre-incubation of resistance arteries with 10 nM ELDP_[162-amide]_ increased the response of 1 nM ET-1 by ≈5 fold (P < 0.002) (Fig. 7B). Consistent with these effects on isolated resistance arteries, ELDP alone (3 nmol/kg) had no effect on blood pressure in anaesthetised rats, but it significantly increased the duration of the pressor response to ET-1 (0.3 nmol/kg) administered 15 min after ELDP (P < 0.02, Fig. 7C) without changing the magnitude of the initial pressor response.

## Discussion

Ever since C-peptide of proinsulin was recognised as a better index of insulin secretion than measuring circulating insulin^39^, research on biomarkers has frequently investigated the utility of other fragments of prohormone precursors. This strategy has been applied successfully to BNP^40^, adrenomedullin^34^, and copeptin (the glycosylated C-terminal peptide of provasopressin)^41^. Previous work has demonstrated the utility of CT-proET-1 measurements^34, 35^, but the precise processing pattern of proendothelin-1 had not been described. Our investigations identified three products of proET-1 synthesis that are co-secreted with ET-1. This has enabled an assessment of these pro-ET-1 peptides as biomarkers in patient samples, as well as investigations of circulating half-lives of these proET-1 peptides in anaesthetised rats to determine whether metabolism has an impact on their utility as biomarkers.

After intravenous injection NT-proET-1 was rapidly eliminated from the circulation (t_½_ < 20 s). Such rapid clearance indicates uptake or metabolism. ET-1 also has a short t_½_ (30–60 s)^42, 43^, which is due primarily to clearance by endothelial ET_B_ receptors in the pulmonary vasculature^44, 45^, and to a lesser extent degradation by neutral endopeptidase 24.11, particularly in post capillary veins^46, 47^. In the absence of receptor-mediated clearance, the high disappearance rate of NT-proET-1 is likely due to peptidase activity in the circulation, particularly in the pulmonary circulation. Bradykinin (RPPGFSPFR) is the archetypal peptide cleared in transit through the lung (t_½_ < 20 s), primarily by angiotensin converting enzyme (ACE)^48, 49^. The kinetics of NT-proET-1 clearance are consistent with first pass metabolism in the pulmonary vasculature, where ACE may well be responsible as it has high affinity for proline-rich sequences^49–51^, which by analogy may result in the NT-proET-1 sequence KPTPSPPWR (ppET-1_[38–46]_) being a susceptible substrate.

In comparison, ELDP and CT-proET-1 had much longer half-lives. Disappearance of stable peptides from the circulation reflects initial dilution in plasma and diffusion into interstitial fluid (phase 1)^52, 53^. This larger volume of distribution, than simple dilution in circulating blood, can account for the lower levels of ELDP and CT-proET-1 at 30 sec than theoretical estimates. Once these peptides have reached distribution equilibrium this is followed by tissue receptor binding, local tissue metabolism or cellular uptake (phase 2), with this second phase being characterised by slower plasma disappearance depending on the tissue clearance mechanisms^52, 53^. The more rapid clearance of ELDP relative to CT-proET-1 may indicate an additional mechanism affecting ELDP such as receptor binding.

Increased levels of ELDP and CT-proET-1 in chronic heart failure may be related to the severity of endothelial dysfunction or to chronic cardiomyopathic changes^54^. The longer circulating t_½_ of CT-proET-1, compared to ELDP, is likely an important factor influencing its higher relative plasma levels. Previous studies describing CT-proET-1 as a biomarker of ET-1 synthesis have been based on an assay for ppET-1_[168–212]_, (i.e. a peptide that includes an additional N-terminal Arg)^25^. Nevertheless, our results are consistent with previous studies showing elevated levels of CT-proET-1 in chronic heart failure^34, 35^, as well as during acute decompensated heart failure^55, 56^. CT-proET-1 is also elevated in acute myocardial infarction, where it was an independent predictor of death and heart failure^57^. Our finding that CT-proET-1 correlates with NT-proBNP and IL-8 suggests that measuring these three biomarkers in combination may be a powerful predictor of outcome in chronic heart failure, or a better index for assessing the effectiveness of new treatments.

The ROC analyses support the superiority of CT-proET-1 over ELDP as a biomarker of chronic heart failure. Similarly, these assays methodologies showed CT-proET-1 levels were a better index of renal dysfunction than ELDP in patients with chronic kidney disease^58^. Whether reduced renal clearance in heart failure or kidney disease affects plasma levels of these peptides is not known. Measurements of CT-proET-1 (ppET-1_[168–212]_) in healthy volunteers showed no significant difference between male (n = 300) and females (n = 218)^59^. However, weak correlations with age and renal function were reported^59^. One of the important limitations of studying metabolism of human peptides in animal models is knowing whether the same pattern of peptidase degradation occurs in the human circulation. Therefore, to extend findings to date, and to provide further validation of CT-proET-1 as a clinical biomarker, infusion studies should be undertaken to define the effect on plasma levels of its clearance rate and metabolism in patients with chronic heart failure, chronic renal failure, and age-matched control subjects. Assays that also detect metabolites of CT-proET-1 may further increase specificity and sensitivity as a diagnostic tool. However, because chronic disease states are frequently associated with increased oxidant stress, an important consideration in biomarker assay development is ensuring amino acid residues that are prone to oxidation are avoided in antigens used for raising antibodies because peptides and metabolites will not be detected if the recognised epitope becomes oxidised^60^. The impact of this has been reported for parathyroid hormone^60^, and we observed His, Met and Trp oxidation in purified peptide fragments we isolated (see Supplementary Information). In addition, a full range of disease states needs to be examined to exclude other potential causes of increased levels such as cancers.

Much of the evidence implicating ET-1 in cardiovascular disease is based on reported increases in tissue expression. However, global overexpression of *EDN1* (ET^+/+^) in mice results in significantly lower blood pressure compared to wild-type animals^61^. This has been attributed, at least in part, to kidney tubular effects of ET-1^61^. Another factor contributing to this modest reduction in blood pressure with *EDN1* overexpression is likely compensatory changes in vascular nitric oxide synthesis, because knockout of endothelial nitric oxide synthase (eNOS) in ET^+/+^ mice results in a greater increase in blood pressure than eNOS knockout alone^62^. Furthermore, endothelial specific over expression of *EDN1* causes a sustained increase in blood pressure^63^. These findings highlight the important differences in potential outcomes of increased *EDN1* expression depending on tissue specific localisation and whether mechanisms are triggered that offset ET-1 upregulation. Hence, in patients with a degree of endothelial dysfunction, increased *EDN1* expression is more likely to cause vasoconstriction and increased blood pressure^64^. Although the increase in blood pressure in mice with endothelial ET^+/+^ was reversed by ET_A_-antagonist treatment^63^, this is not always the case as the increased blood pressure and arteriolar remodelling when endothelial ET^+/+^ was combined with salt-loading were only partially prevented by ET_A_-antagonist administration^65^. This suggests other peptides derived from proET-1 contribute to the biological effects of *EDN1* expression through yet to be identified receptor mechanisms. Perhaps of most significance is the ERA-resistant heart failure resulting from cardiac over expression of human *EDN1* in mice, where combined ET_A_- and ET_B_-antagonist treatment failed to prevent the lethal effects of this transgenic modification^7^. Our results indicate a potential role for ELDP in disease processes by increasing the magnitude of ET-1 responses, but further investigation is required to determine whether ELDP contributes to ERA-resistant effects of *EDN1* overexpression.

An important component of ET-1-induced vasoconstriction is Ca^2+^ influx through voltage-gated L-type Ca^2+^ channels^66, 67^. Modulation of ET-1 responses by ELDP therefore likely involves facilitation of Ca^2+^ influx. Responses to norepinephrine and serotonin are also augmented by ET-1, and this has been attributed to increased Ca^2+^ influx^68–70^. To investigate whether ELDP mediates its effects via a distinct G-protein coupled receptor (GPCR), whose ligand pairing had yet to be assigned, ELDP was evaluated against a panel of cell-expressed orphan GPCRs using a detection system based on ß-arrestin binding (Supplementary Figure S3). This screen failed to identify an ELDP receptor – ligand pair. Negative results could be due to: the relevant GPCR not being included in the test panel, inadequate stability of ELDP during incubations, or failure to induce receptor binding of ß-arrestin because of G-protein biased receptor signalling^71, 72^. Alternatively, ELDP responses may be mediated through a mechanism distinct from GPCR activation.

Vascular smooth muscle P2X_1_ receptors represent a good example of non-GPCR modulation of vasoconstrictor responses^73^. P2X_1_ receptor responses are similar to those of ELDP as P2X_1_ stimulation has little effect in the absence of vascular tone, but it potentiates other vasoconstrictors including ET-1^74, 75^. Activation of this ligand-gated ion channel by ATP leads to Ca^2+^ influx and membrane depolarization, which causes further Ca^2+^ entry via voltage-gated Ca^2+^ channels and lowers the threshold for vasoconstriction^73^. ELDP-induced vasoconstriction following partial pre-constriction with ET-1 is consistent with a ligand-gated ion channel reducing the threshold for vasoconstriction and augmenting ET-1 induced Ca^2+^ entry^73–75^. Hence, the receptor target for ELDP may be an ion channel with similar properties to the P2X_1_ receptor. Certainly, the high degree of sequence conservation for ELDP during evolution is likely due to the structural constraints of a specific receptor target. Ligand-gated ion channels show a high degree of evolutionary conservation^76, 77^, but the pharmacology, including ligands activating some of these family members, has yet to be fully investigated^77^, and may be relevant receptor targets for ELDP. Modulation of ET-1 responses by ELDP also has implications for treatment of non-cardiovascular diseases, such as cancer and fibrosis, where ET_A_-receptor antagonists are generally ineffective despite strong evidence that *EDN1* expression is involved in disease progression^14^. Moreover, ligand-gate ion channels have been implicated in cancer cell proliferation^78^.

In summary, our investigations identified three fragments generated by intracellular processing of proET-1 – NT-proET-1, ELDP and CT-proET-1. These peptides are co-secreted with ET-1 and closely correlate with ET-1 synthesis. Assessment of these peptides as biomarkers confirmed previous investigations demonstrating CT-proET-1 has the greatest potential as a diagnostic tool for detecting *EDN1*-linked pathologies. Furthermore, evaluation of the systemic clearance of these proET-1 peptides in anaesthetised rats has provided a physiological basis for the higher relative levels of CT-proET-1. Identification of ELDP as the evolutionary conserved proET-1 fragment containing the endothelin-like domain has enabled an initial examination of its biological activity. This indicates the potential for ELDP to have pathophysiological effects, which merit further investigation.

## Materials and Methods

### Synthetic peptides

Peptide sequences of proET-1 peptides are shown in Fig. 1. NT-proET-1 (ppET-1_[18–50]_), ELDP (ppET-1_[93–166]_) and CT-proET-1 (ppET-1_[169–212]_) were chemically synthesised using Boc chemistry on a modified ABI 433 synthesiser as described^79^. ELDP was produced by total chemical synthesis using native chemical ligation to link an N-terminal fragment (residues 1–30; ppET-1_[93–122]_) with a C-terminal fragment (residues 31–74; ppET-1_[123–166]_) containing an N-terminal Cys residue^80^. Refolding of ELDP and formation of disulphide bridges was carried out overnight by stirring a solution of the peptide (0.1 mg/ml) in 0.1 M Tris HCl pH 8.0 with 2 M guanidinium chloride (G-HCl) and reduced/oxidised glutathione 0.5/0.1 mM. The following day the solution was acidified and purified by reverse-phase chromatography. ELDP_[162-amide]_ (ppET-1_[93–162-amide]_) was synthesised following the same approach with a C-terminal fragment of residues 31–70 (ppET-1_[123–162]_ with C-terminal arginine-amide). Purity of all peptides was confirmed by HPLC and mass spectrometry.

### ProET-1 antibodies and immunoassays

Underlined sequences in Table 1 indicate peptide antigens used to raise specific antisera for sandwich immunoassays following previously described methodologies^30^. Immunogens for ppET-1_[93–109]_ and ppET-1_[155–166]_ were prepared by conjugation via terminal Cys residues to maleimide groups on pre-treated carrier proteins^30^. To enable conjugation of ppET-1_[18–30]_, ppET-1_[93–102]_ and ppET-1_[169–186]_ by the maleimide methodology, synthetic peptides for these antigens included a C-terminal Cys-NH_2_ residue. Immunogens for ppET-1_[42–50]_ and ppET-1_[204–212]_ were prepared by glutaraldehyde conjugation to carrier protein^30^. For initial characterisation of conditioned media samples, rabbit antisera raised against ppET-1_[42–50]_ and ppET-1_[93–102]_ were used to establish radioimmunoassays for these peptide antigens. Subsequent investigations used sandwich ELISA methodologies with specific IgG purified from antisera using the respective peptide antigens (Table 1) coupled to SulfoLink Coupling Resin (Thermo Scientific/Pierce) or CNBr-Sepharose (GE Healthcare)^30^. Purified IgG for use as detection antibodies were biotinylated with NHS-LC-LC-biotin (Thermo Scientific/Pierce), and used for assay measurements with enhanced chemiluminescence^30^.

A multiplexed immunoassay system was used for simultaneous measurement of NT-proET-1, ELDP and CT-proET-1 in small volumes of rat plasma. Capture antibodies were conjugated to magnetic beads (region 45 – NT-proET-1, region 36 – ELDP, and region 27 – CT-proET-1) following standard protocols (Luminex Corporation). For assay capture antibody-coated magnetic beads were diluted as a premixed combination (10 µl/well) and incubated overnight at 4 °C on an orbital plate mixer with plasma (15 µl) and assay buffer (135 µl). For detection, beads were washed using a ring magnet plate washer before adding 25 µl of combined biotinylated detection antibodies and incubating for 2 h at room temperature on an orbital plate mixer. Detection was achieved with streptavidin-phycoerythrin (25 µl/well) incubated for 30 min at room temperature before reading on a Luminex 200 System.

### Cell Culture

EA.hy 926 and A549 cell lines were grown in Dulbecco’s modified Eagle medium with 10% fetal bovine serum at 37 °C in a humidified CO_2_ incubator (8% CO_2_, 92% air) as previously described^28, 30^. Human aortic endothelial cells were passaged cells from primary cultures grown in endothelial cell basal medium with growth supplement (Cell Applications Inc.), and used when confluent to compare secretion of proET-1 peptides with ET-1. Purified apple procyanidin pentamer was from Asahi Breweries (Moriya, Ibaraki 302–0106, Japan), grape seed extract was form Polyphenolics (Madera, CA), TNFα and TGFβ were from R&D Systems.

### Characterisation and purification of proET-1 peptides

For initial characterisation of proET-1 processing, confluent cultures of EA.hy 926 and A549 cell lines were incubated for 48 h with serum-free DMEM (30 ml/T-175 cm^2^ flask) containing peptidase inhibitors (1 mM bacitracin, 10 µM phosphoramidon, 10 µM leupeptin, 10 µM chymostatin and 1 µM pepstatin A). Proendothelin-1 peptides were isolated by precipitation with ammonium sulphate (90% saturation at 4 °C) and characterised by size exclusion chromatography (Fig. 2A and B).

For purification of proET-1 peptides for LC-MS/MS characterisation, serum free conditioned media was collected after 48 h incubation with confluent cultures of EA.hy 926 cells in T175 cm^2^ flasks (25 ml media/flask) was acidified with 1.25% CH_3_COOH and centrifuged at 3,000 rpm at 4 °C for 15 min to remove insoluble material. Acidified samples (30 ml/column) were passed through mini columns of Q-Sepharose Fast Flow gel (GE Healthcare) (6 ml bed volume × 10 columns per batch of conditioned media) pre-equilibrated with 10 mM acetic acid. Unadsorbed peptides were then loaded onto Fractogel CEx weak cation exchange columns (2.8 ml bed volume × 10 columns) (Fractogel EMD COO^−^ 650 M; E.Merck) (Fig. 2C). Prior to HPLC eluted fractions (Fig. 2C) containing ELDP and CT-proET-1 from Fractogel CEx were desalted by solid phase extraction on C2 silica cartridges (500 mg, Waters) equilibrated with 0.1% TFA, and eluted with 80% CH_3_CN containing 0.1% TFA. NT-proET-1 was not adsorbed on Q-Sepharose FF gel or Fractogel CEx, and was recovered from the eluate passing through both columns by solid phase extraction on C2 silica cartridges.

ProET-1 peptides were subjected to reverse phase HPLC on C4 silica (15 µm, 300 Å pore, 1 × 25 cm; Jupiter^®^ Phenomenex) with gradient elution at 2 ml/min: 0.1% TFA – 15% solvent B (80% CH_3_CN with 0.1% TFA) over 2 min, followed by 15–60% B over 45 min. Fractions (1 min) were collected for sandwich immunoassay of NT-proET-1, EDLP and CT-proET-1. Peak fractions for each peptide were separately purified using higher resolution reverse phase HPLC on C4 silica (5 µm, 300 Å pore, 4.6 × 250 mm; ACE-5 C4–300, Advanced Chromatography Technologies) (Fig. 2D,E and F). Synthetic peptides were used to characterise elution positions of NT-proET-1, ELDP and CT-proET-1 on this second HPLC system by monitoring absorbance at 280 nm and immunoassay of fractions. To avoid contamination by synthetic peptides these peptide standards were only run after HPLC of purification of the native peptides.

### LC-MS/MS characterisation

The identities of purified ELDP and CT-proET-1 were confirmed by LC-MS/MS after tryptic digestion (without reduction and alkylation). HPLC fractions containing the highest immunoreactivity for ELDP and CT-proET-1 were dried before digesting with sequence-grade trypsin 0.5 µg/µl at a final trypsin:protein ratio of 1:50 in 20 mM ammonium bicarbonate buffer, pH 7.8 at 37 °C overnight (18 h). Tryptic peptides were separated by nanoflow UPLC using a C18 column (5 µm, 180 µm × 20 mm; Symmetry, Waters) eluted with 0.1% formic acid and a gradient of acetonitrile (1–35% over 10 min), and analysed using an LTQ-Orbitrap XL MS equipped with a nano-ESI source (positive mode) (Thermo Fisher Scientific). Full scan survey spectra (m/z 375–1800) were acquired with a resolution of 30,000 at m/z 400.

Purified NT-proET-1 was analysed in its intact form by nanoACQUITY UPLC using a C4 column (1.7 µm, 300 Å, 75 µm × 100 mm; BEH300 Waters) by gradient elution at 300 nl/min with 0.1% formic acid and a gradient of acetonitrile 5–50% over 25 min and analysed by LTQ-Orbitrap XL MS.

Raw data from MS/MS was subjected to database search using MASCOT (Matrix Science Ltd). In addition, theoretical ion fragments from trypsin digestion of ELDP and CT-proET-1 were obtained from ProteinProspector (http://prospector.ucsf.edu/) for the endothelin-1 gene *EDN1* (P05305, http://www.uniprot.org/uniprot/P05305), and raw chromatograms were subsequently analysed using Xcalibur. Fragment ions were identified in the MS/MS spectra from Qual Browser and labelled manually for the b- and y-ions, confirming MS/MS fragmentation from MASCOT. All samples were run twice on two separate days and only peptides that were present in all analyses are described here (see Supplementary Tables S1 and S2, Supplementary Figures S1 and S2).

### Evaluation of proET-1 peptides as biomarkers of heart disease

ELDP and CT-proET-1 were measured using sandwich ELISAs with chemiluminescence detection in plasma samples from patients with stable chronic heart failure (HF, n = 24)^54^, and compared with untreated control subjects with pre-hypertension/mild hypertension that were free of any symptoms of ischemic heart disease or heart failure (HYP, n = 24)^81^. Blood samples from patients with stable chronic heart failure were collected at the London Chest Hospital, Barts Health NHS Trust, London, UK with the approval of the local research ethics committees and written informed consent of each subject (East London and the City Research Ethics Committee – reference number 07/Q0604/24)^54^, and from untreated subjects with pre-hypertension/mild hypertension were collected in the Centre for Clinical Pharmacology, William Harvey Research Institute, Charterhouse Square, London, UK (East London and the City Research Ethics Committee – reference number 07/Q0605/44)^81^. These investigations were performed in accordance with the relevant guidelines and regulations. Subject demographic data are summarised in Table 2. CRP was measured by ELISA (MP Biomedicals). IL-6, IL-8, MCP-1 and TNFα were measured in samples from with patients with chronic heart failure by multiplex assay technology (CVD panel 2 – inflammation, Novagen/Merck Chemicals) read on a Luminex 200 System.

### Animal studies

All experiments were conducted according to the Animals (Scientific Procedures) Act 1986, UK, and approved by the UK Home Office. Clearance rates of proET-1 peptides and blood pressure effects of ELDP were determined in male Wistar rats (Charles River) anaesthetised with sodium thiopentone (120 mg/kg i.p.). Peptides were administered *i.v*. via a polyethylene catheter in the left femoral vein. Blood samples (0.5 ml) were collected from a catheter in the left carotid artery into chilled 1.5 ml microcentrifuge tubes containing 50 U/5 µl of heparin. Samples were centrifuged at 12,000 rpm for 2 min at 4 °C. Plasma (50 µl aliquots) samples were stored at −80 °C for subsequent analyses. Blood pressure and heart rate were recorded from the left carotid artery via a polyethylene catheter using Labchart 7.1 (AD Instruments Ltd).

Vasoconstrictor actions of ELDP and interactions with ET-1 were investigated using rat isolated mesenteric resistance arteries mounted in an automated tension myograph (Danish Myo Technology, Denmark) following previously described methods^82, 83^. Contractile responses are expressed as a percentage of the maximal responses to phenylephrine (10 µM).

### Statistical analyses

Data were analysed using GraphPad Prism 6.0 (GraphPad Software Inc.). Results are shown as mean ± SD except for studies of cardiovascular effects which are mean ± SEM. Statistical differences were determined by analysis of variance with post hoc Bonferroni multiple comparisons test, or by t-test for two group comparisons. Plasma values from patients with chronic heart failure were compared to disease free controls using ROC (Receiver Operating Curve) analyses, with difference between areas under the curve (AUC) assessed using StAR software^84^. ROC analyses were adequately powered for a phase 1/exploratory assessment of a diagnostic biomarker^85^.

### Data availability

All data generated or analysed during this study are included in this published article (and its Supplementary Information files).

## Electronic supplementary material

Supplementary Information
