# Supplementary material for: Characterisation of preproendothelin-1 derived peptides identifies Endothelin-Like Domain Peptide as a modulator of Endothelin-1
Source: Sci Rep. 2017 Jul 10;7:4956. doi: 10.1038/s41598-017-05365-2 (PMC5503984; doi:10.1038/s41598-017-05365-2)
Supplement: Supplementary file 1 — Supplementary Information [file 41598_2017_5365_MOESM1_ESM.pdf]

Supplementary data for

Characterisation of preproendothelin-1 derived peptides identifies Endothelin-Like Domain Peptide as a modulator of Endothelin-1

Jale Yuzugulen, Julie A. Douthwaite, Elizabeth G. Wood, Inmaculada C. Villar, Nimesh S.A. Patel, James Jegard, Hubert Gaertner, Irène Rossitto-Borlat, Keith Rose, Oliver Hartley, Pedro R. Cutillas, Amrita Ahluwalia & Roger Corder

Supplementary Table S1: MASCOT identification of tryptic peptides from purified native ELDP – HPLC fractions 43 (A) and 47 (B). *N- and C-terminal residues corresponding to the sequence of ppET-1 (ELDP; ppET-1$_{[93-166]}$), monoisotopic and experimental average masses, charge, score and identified peptide are indicated.* ***H**, **M** and **W** indicate oxidation of the corresponding His, Met and Trp residues. **N** indicates deamidation of asparagine. Bold m/z values correspond to peptide ions selected for MS/MS to provide data for primary structure determination (Figure S1).*

(A) Purified native ELDP, fraction 43:

ppET-1	m/z (Da)	Average Mass	Charge	Score	Peptide
128–137	**625.308**	1,248.602	2	44	ELRAEDI**M**EK
128–143	681.990	2,042.948	3	26	ELRAEDI**M**EKDWNNHK
	511.744	2,042.948	4	12	ELRAEDI**M**EKDW**N**NHK
131–137	426.195	850.375	2	26	AEDI**M**EK
131–143	823.368	1,644.721	2	61	AEDI**M**EKDWNNHK
	549.247	1,644.721	3	36	
131–144	**887.414**	1,772.814	2	43	AEDI**M**EKDWNNHKK
	591.942	1,772.805	3	26	AEDI**M**EKDW**N**NHKK
	597.277	1,788.810	3	35	AEDI**M**EKD**W**NNHKK
155–162	**540.287**	1,078.560	2	49	CIYQQLVR

(B) Purified native ELDP, fraction 47:

ppET-1	m/z (Da)	Average Mass	Charge	Score	Peptide
128–137	617.310	1,232.605	2	34	ELRAEDIMEK
	625.308	1,248.602	2	50	ELRAEDI**M**EK
128–143	676.657	2,026.950	3	47	ELRAEDIMEKDWNNHK
	681.990	2,042.948	3	21	ELRAEDI**M**EKDWNNHK
	687.321	2,058.942	3	21	ELRAEDI**M**EKD**W**NNHK
131–137	418.197	834.380	2	47	AEDIMEK
	426.194	850.374	2	26	AEDI**M**EK
131–143	815.369	1,628.723	2	34	AEDIMEKDWNNHK
	543.915	1,628.724	3	28	
	549.247	1,644.719	3	39	AEDI**M**EKDWNNHK
	823.367	1,644.719	2	35	
	554.579	1,660.716	2	23	AEDI**M**EKDWNN**H**K
131–144	879.417	1,756.820	2	56	AEDIMEKDWNNHKK

	586.61	1,756.808	3	50	
	887.415	1,756.815	2	46	AEDI**M**EKDWNNHKK
	591.943	1,772.808	3	28	
	591.945	1,772.814	3	33	AEDIMEKDWNN**H**KK
	597.606	1,789.795	3	23	AEDI**M**EKD**W**N**N**HKK
155–162	540.287	1,078.559	2	49	CIYQQLVR

Supplementary Figure S1A

(A)

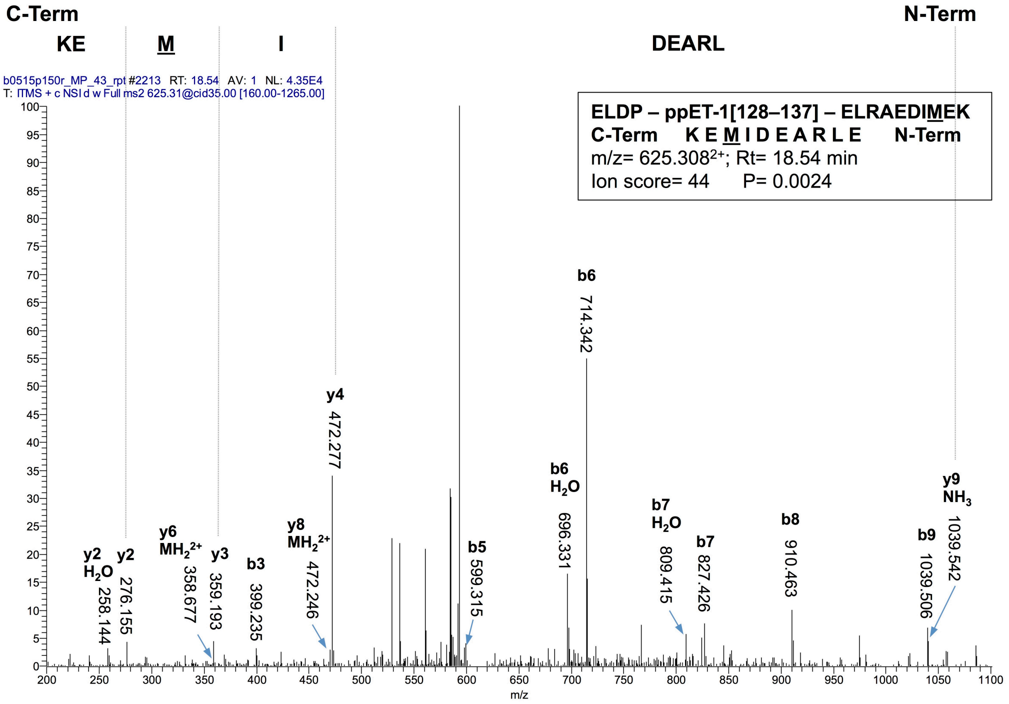

Supplementary Figure S1B

(B)

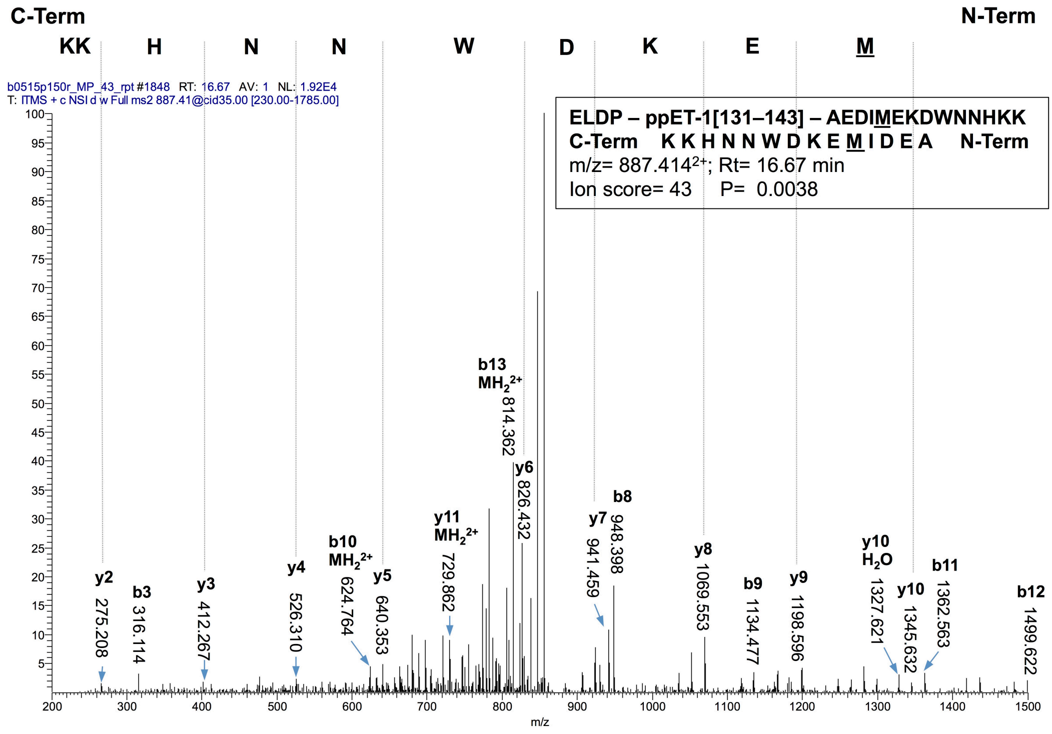

Supplementary Figure S1C

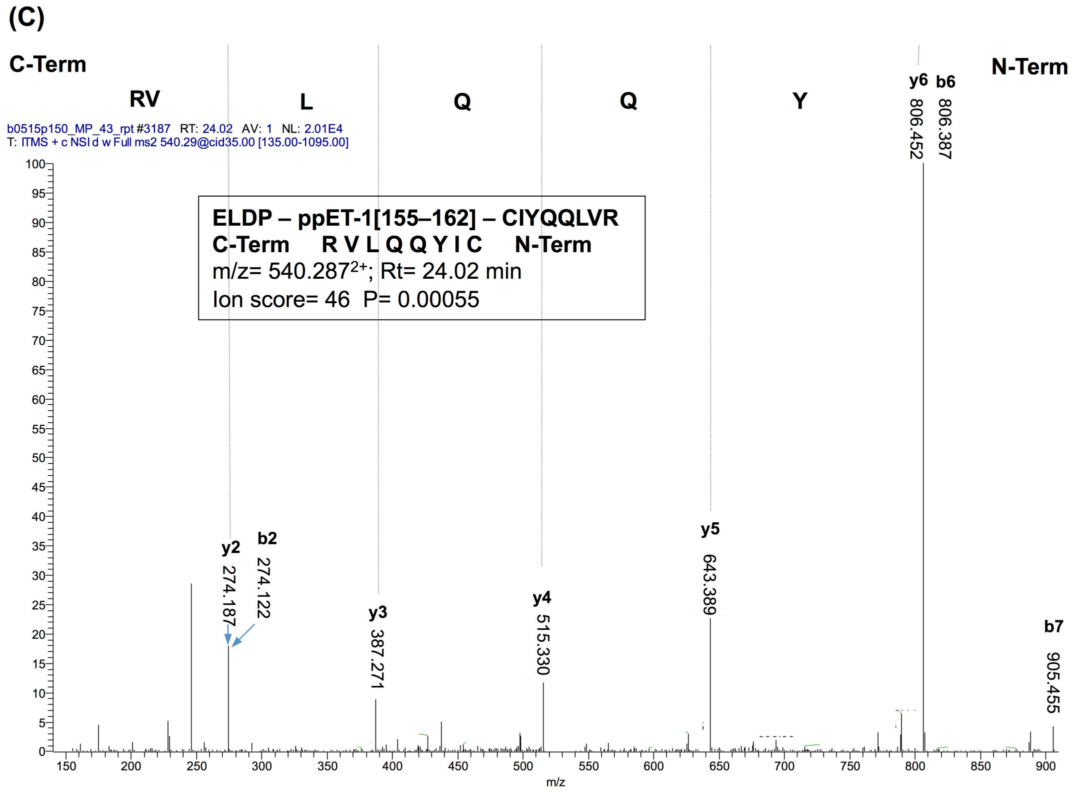

Supplementary Figure S1: MS/MS spectra of purified native ELDP (fraction 43) precursor ions at m/z (A) 625.308^{2+}, (B) 887.414^{2+} and (C) 540.287^{2+}. *MS/MS spectra were manually annotated for C-terminal fragment ions (y-) and N-terminal fragment ions (b-) confirming the sequence identities of the precursor ions obtained from MASCOT. The mass differences between the y-ion series indicate amino acid residues of the corresponding fragment ions, which are shown above the spectrum. Annotations of – NH_3 or H_2O at b- and y-ions represent a loss of ammonia or water from the corresponding fragment ions with a loss of 17 and 18 Da, respectively*

Supplementary Table S2: MASCOT identification of tryptic peptides from purified native CT-proET-1 – HPLC fraction 28. *N- and C-terminal residues corresponding to the sequence of ppET-1 (CT-proET-1; ppET-1$_{[169–212]}$), monoisotopic and experimental average mass, charge, score and identified peptide are indicated.* ***M*** *indicates oxidation of the corresponding methionine. Bold m/z values correspond to peptide ions selected for MS/MS to provide data for primary structure determination (Figure S2).*

ppET-1	m/z (Da)	Average Mass	Charge	Score	Peptide
169–183	**461.735**	1,842.911	4	2	SSEEHLRQTRSETMR
179–187	**534.261**	1,066.508	2	23	SET**M**RNSVK
184–196	496.273	1,485.796	3	20	NSVKSSFHDPKLK
188–196	529.786	1,057.557	2	30	SSFHDPKLK
188–201	**528.627**	1,582.86	3	60	SSFHDPKLKGKPSR
204–209	**395.204**	788.393	2	12	YVTHNR

Supplementary Figure S2A

(A)

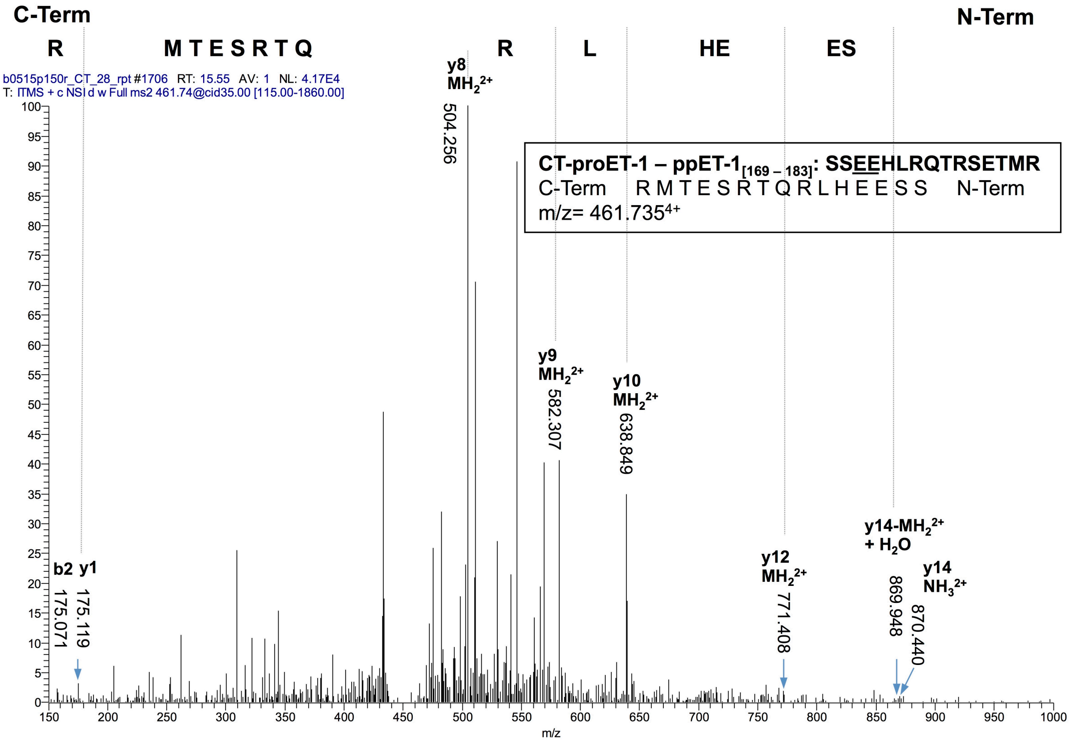

Supplementary Figure S2B

(B)

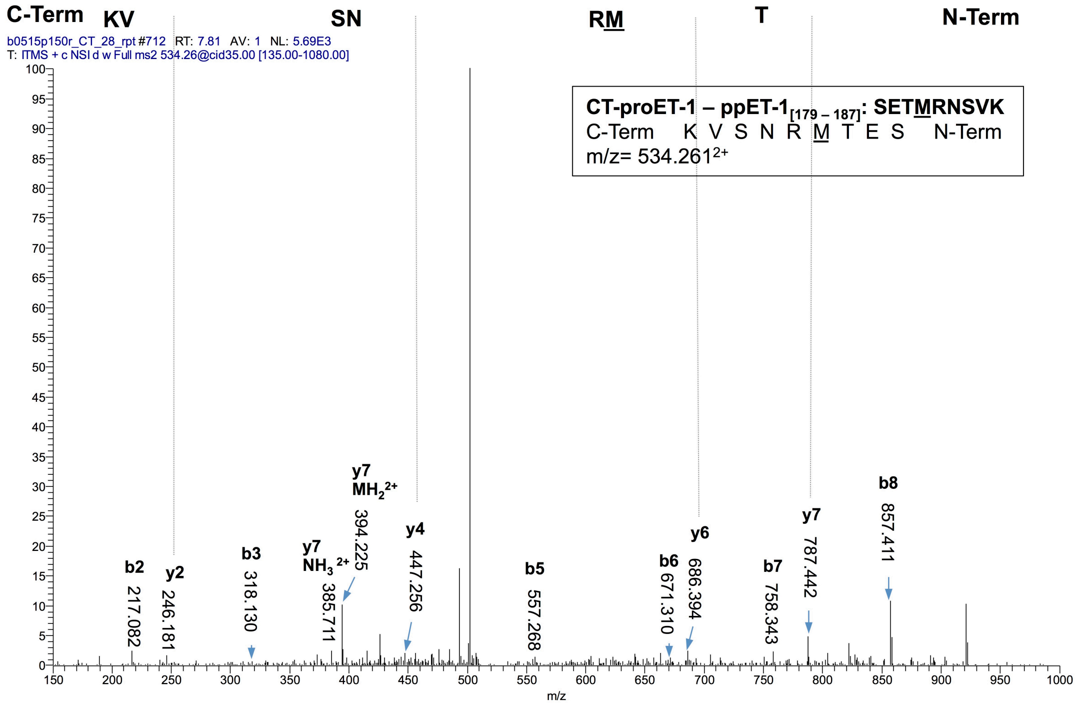

Supplementary Figure S2C

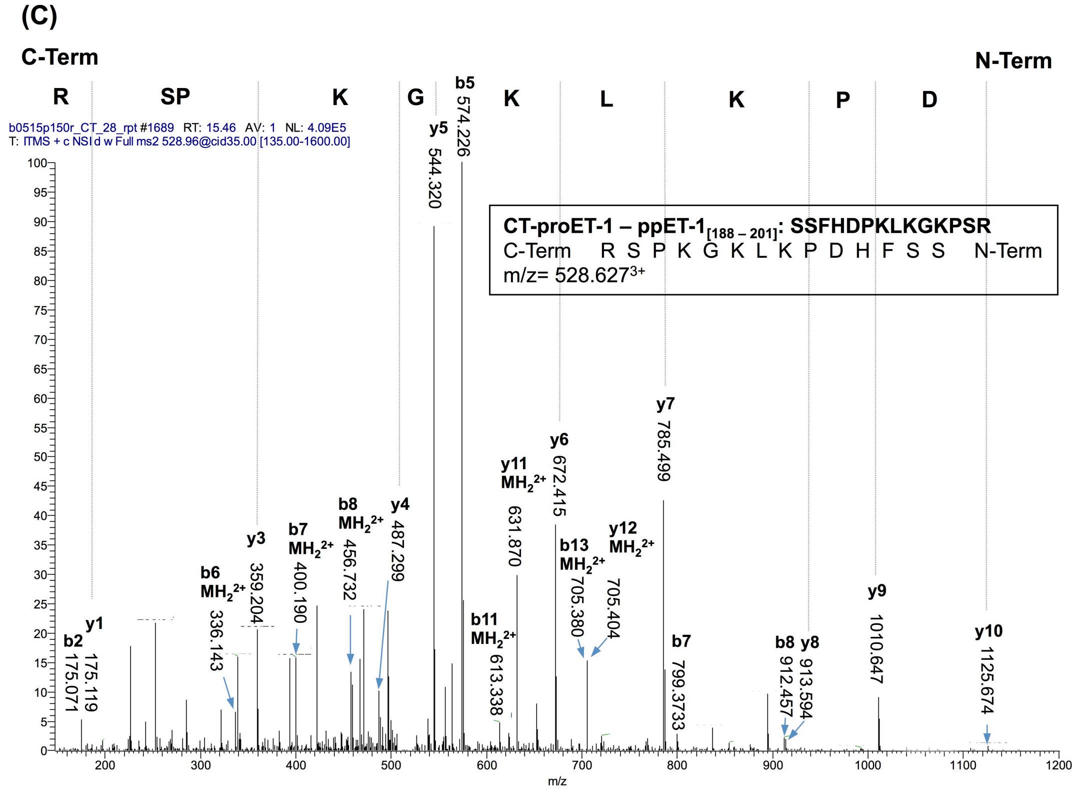

Supplementary Figure S2D

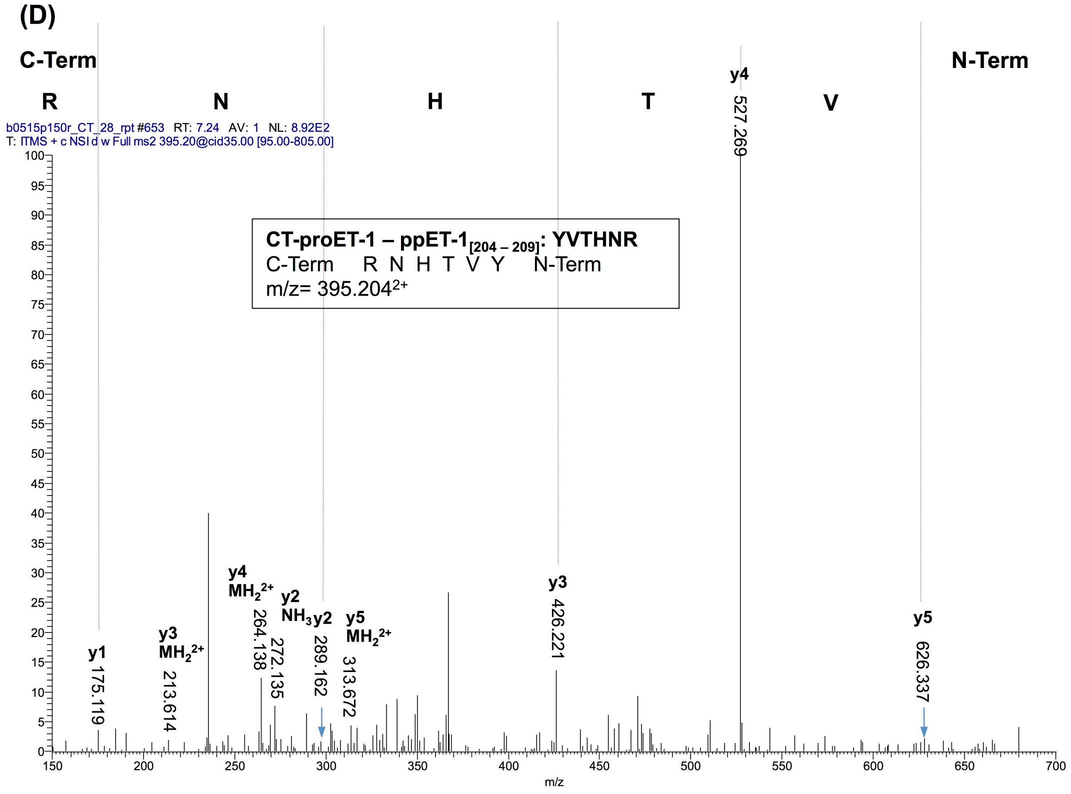

Supplementary Figure S2: MS/MS spectra of purified native CT-proET-1 (fraction 28) precursor ions at m/z (A) 461.735^{4+}, (B) 534.261^{2+}, (C) 528.627^{3+} and (D) 395.204^{2+}. *MS/MS spectra were manually annotated for C-terminal fragment ions (y-) and N-terminal fragment ions (b-) confirming the sequence identities of the precursor ions obtained from MASCOT. The mass differences between the y-ion series indicate amino acid residues of the corresponding fragment ions, which are shown above the spectrum. Annotations of –* NH_3 *or* H_2O *at b- and y-ions represent a loss of ammonia or water from the corresponding fragment ions with a loss of 17 and 18 Da, respectively.*

Supplementary Figure S3 - ORPHAN G-PROTEIN-COUPLED RECEPTORS SCREENED WITH ELDP

The PathHunter Orphan GPCR biosensor panel (DiscoverRx Corporation, Fremont, CA 94538, USA) was used to screen for receptor – ligand pairing of ELDP (1,2). The principle of this cell-based methodology is an enzyme fragment complementation system where receptor coding sequences are fused with a ß-galactosidase coding fragment and expressed in cells with a ß-arrestin coding sequence fused to a second ß-galactosidase coding fragment. On binding of ligand to the cell expressed receptor, ß-arrestin binds to the activated GPCR, this results in the ß-galactosidase fragments attached to ß-arrestin and to the GPCR complementing each other to reconstitute ß-galactosidase activity, which is then measured by chemiluminescence ß-galactosidase assay as an index of receptor binding (1,2).

EXPERIMENTAL DETAILS

Orphan GPCR screening for ELDP was undertaken by DiscoverRx Corporation, Fremont, CA 94538, USA. ELDP (0.1 μM) was incubated for 90 min at room temperature in duplicate in 384-well format with 5000 cells per well expressing the GPCRs indicated. Control (PBS vehicle) incubations were performed in parallel. At the end of the incubation period chemiluminescent substrate was add, and incubated for 60 min before measurement of relative luminescence (RLU) using a PerkinElmer Envision^{TM} instrument.

Data Analysis: Percentage activity was calculated for each test well relative to the mean RLU of the corresponding vehicle-treated GPCR. Results are expressed as mean control (%) ± coefficient of variation (%).

RESULTS

Compared to vehicle control cells none of the orphan GPCRs showed a significant increase in activity after incubation with ELDP (supplementary Figure S3). Also, no responses exceeded the benchmark for a positive signal, which is inferred by an increase in ß-galactosidase activity that exceeds three standard deviations of the respective control value.

REFERENCES

1. Yan, Y. X., Boldt-Houle, D. M., Tillotson, B. P., Gee, M. A., D'Eon, B. J., Chang, X. J., Olesen, C. E., Palmer, M. A. (2002) Cell-based high-throughput screening assay system for monitoring G protein-coupled receptor activation using beta-galactosidase enzyme complementation technology. *J. Biomol. Screen.* **7**, 451–459

2. Yin, H., Chu, A., Li, W., Wang, B., Shelton, F., Otero, F., Nguyen, D. G., Caldwell, J. S., Chen, Y. A. (2009) Lipid G protein-coupled receptor ligand identification using beta-arrestin PathHunter assay. *J. Biol. Chem.* **284**, 12328–12338

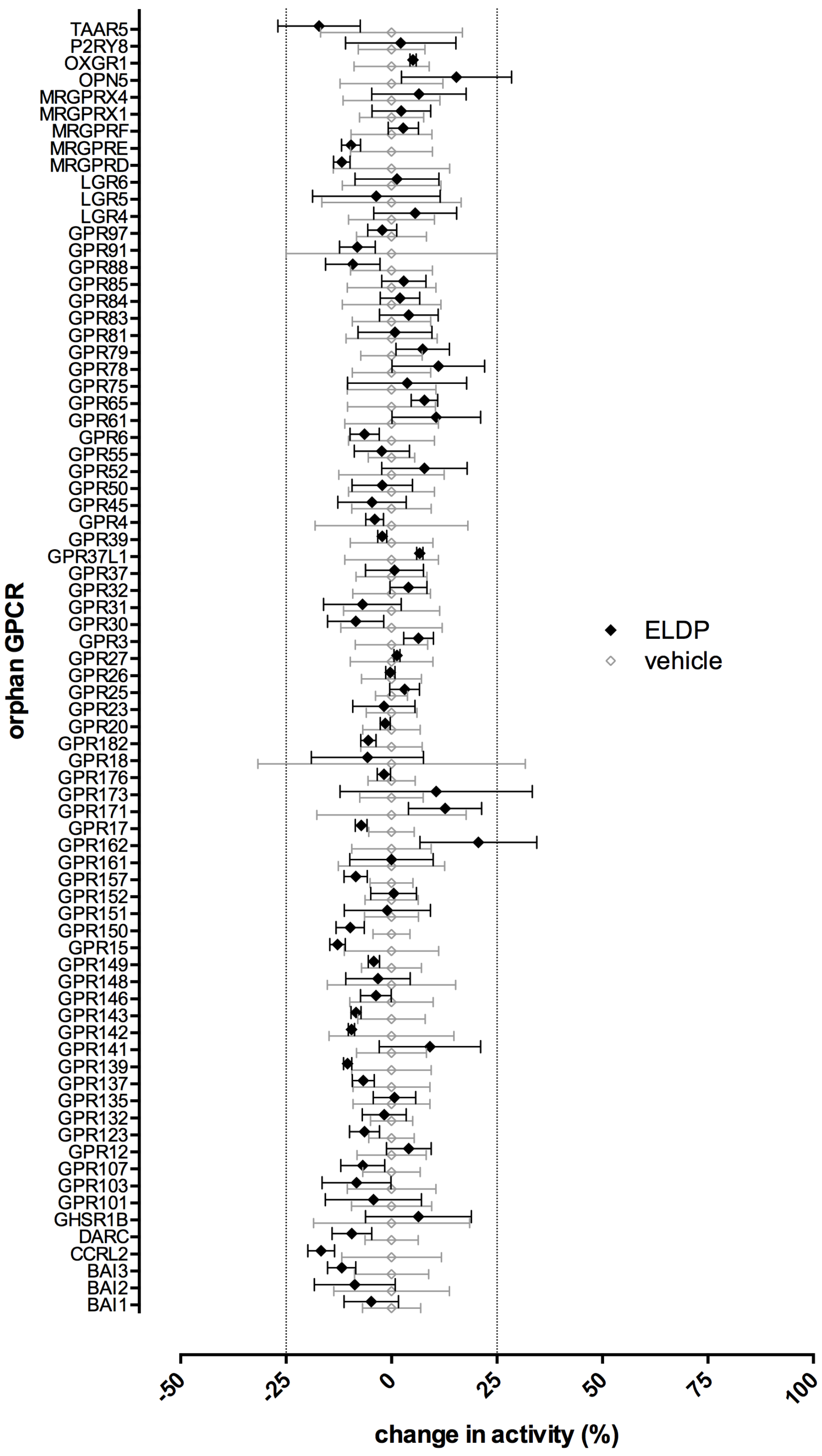

Supplementary Figure S3: Orphan GPCR screen for ELDP. *Change in β-galactosidase activity after incubation with 0.1µM ELDP (mean ± CV%) compared to corresponding control incubations.*
